# Editorial: “Natural Products as a Tool to Design New anti-MDR Lead Molecules.”

**DOI:** 10.3389/fphar.2021.694674

**Published:** 2021-06-21

**Authors:** Patrícia Rijo, Francisco Estévez, Przemysław Sitarek, Maria-José U. Ferreira

**Affiliations:** ^1^ CBIOS—Universidade Lusófona´s Research Center for Biosciences & Health Technologies, Lisboa, Portugal; ^2^ Research Institute for Medicines (iMed.ULisboa), Faculty of Pharmacy, Universidade de Lisboa, Lisboa, Portugal; ^3^ University of Las Palmas de Gran Canaria, Las Palmas de Gran Canaria, Spain; ^4^ Department of Biology and Pharmaceutical Botany, Medical University of Lodz, Lodz, Poland

**Keywords:** traditional medicines, herbal medicines, natural products, MDR, cancer

Natural products have been used in traditional medicine, since ancient times, for the treatment of several diseases. Characterized by a high structural diversity, they have long represented an important source of medicines or drug leads, playing a key role in drug discovery and development, namely in cancer. Currently, there is great interest in identifying small molecules from natural sources for overcoming multidrug resistance (MDR), a major obstacle in cancer treatment, by restoring the efficacy of chemotherapeutic agents, preventing MDR, or re-sensitizing tumour cells to anticancer drugs.

This special issue collects ten contributions, two reviews and eight original articles, highlighting the importance of natural products as source of new anti-MDR lead molecules in cancer.

A review article by Ferreira et al. focused on the disruption of nuclear and cell protein nuclear exports *via* CRM1, which has become a promising therapeutic strategy for the treatment of patients with viral infections and cancer. The clinical approval of the first-in-class CRM1 inhibitor, Selinexor, has demonstrated the therapeutic potential of inhibiting CRM1 in cancer treatment. Compounds derived from natural sources hold promise to support the discovery of new nuclear export inhibitors with a novel mode of action.

The review work by Tinoush et al. summarized reports of several secondary metabolites, with different scaffolds, capable of reversing MDR in cancer by inhibiting the transport activity or the expression of P-gp, MRP1, and BRCP, which are the three main ABC transporters implicated in MDR. Moreover, synergistic interactions of different natural product-derived compounds, in combination with anticancer drugs, were also addressed. Although the efficacy of phytochemicals requires clinical confirmation, there are many studies providing evidence for their promising anti-MDR role, being, currently, considered as 4th generation ABC transporter modulators.

Changes of cancer cells responsible for resistance to anticancer drugs might, at the same time, generate vulnerabilities that may rend drug-resistant cells more sensitive to other compounds, named collateral sensitivity agents (CS). Using this anti-MDR approach, Reis et al. evaluated the CS effect of a set of macrocyclic diterpene derivatives of the lathyrane-type against several human cancer cell lines and corresponding drug-resistant sub-lines. Some compounds were found to be promising CS agents, being many-fold more effective against MDR sublines, mainly in relation to gastric carcinoma cells. These compounds were able to induce apoptosis *via* caspase-3 activation. Structure-activity relationships of the compounds were also obtained through regression models.

In turn, Liao et al. attempted to elucidate whether the combination of tanshinone IIA and cisplatin could generate a synergistic antitumor effect on esophageal squamous cell carcinoma (ESCC) cells. The authors revealed that the combination suppressed cell migration and invasion abilities, arrested the cell cycle, and induced apoptosis in HK and K180 cells. Molecular docking studies indicated that tanshinone IIA and cisplatin could be docked into active sites with the tested proteins. Additionally, the expression levels of E-cadherin, β-catenin, Bax, cleaved caspase-9, P21, P27, and c-Fos were upregulated, and the expression levels of fibronectin, vimentin, Bcl-2, cyclin D1, p-Akt, p-ERK, p-JNK, P38, COX-2, VEGF, IL-6, NF-κB, and c-Jun proteins were downregulated.

He et al. work reported that dioscin promoted apoptosis of prostate cancer cells and inhibited cell invasion by increasing SHP1 phosphorylation and suppressing the downstream MAPK signalling pathway. The results of *in vitro* and *in vivo* studies have showed that dioscin reversed IL-6 and DHT-stimulated PCa cell proliferation and invasion and increased apoptosis in these cells. The anticancer activity of dioscin may be due to its effects of increasing SHP1 phosphorylation and inhibiting the subsequent MAPK signalling pathway. Therefore, the results suggested that dioscin may be a potential drug to treat both androgen-sensitive and androgen-independent PCa.

Liu et al. reported the phytochemical study of the traditional Chinese medicine Marsdeniae tenacissimae Caulis, whose constituents were submitted to cytotoxicity screening, pharmacology analysis, and cellular and molecular experiments. The study showed that four C21 steroidal saponins were able to induce apoptosis in lung adenocarcinoma (A549), by downregulating the anti-apoptotic protein Bcl2 and upregulating the pro-apoptotic protein Bax. In addition, these compounds also downregulated the expression of MMP-2 and MMP-9 proteins, thus providing evidence for the potential of Marsdeniae tenacissimae Caulis against lung cancer.

AlQathama et al. studied the influence of selected Nigerian medicinal plants on the viability, mobility, and resistance mechanisms in liver, colon and skin cancer cell lines. They demonstrated that some of herbal medicines were endowed with significant *in vitro* cytotoxicity and inhibited MDR mechanisms. The aqueous extracts of *Gongronema latifolium* and *Strophanthus hispidus* were the most cytotoxic against Caco2 cells. In turn, *Bridelia ferruginea*, and *Ximenia americana* extracts showed the strongest inhibition of P-gp efflux activity. Moreover, inhibitory effects on other MDR mechanisms, namely depletion of glutathione, were also observed for some extracts, whereas others exerted *in vitro* anti-migratory activity against the highly metastatic B16-F10 cell line.

Vágvölgyi et al. unveiled two new ecdysteroid squalenylated prodrugs that were characterized and formulated as self-assembling nanoparticles. The authors revealed that, in the checkerboard microplate assay, one of the starting ecdysteroids showed strong synergism with doxorubicin, in multidrug resistant mouse lymphoma cells, whereas the other compound showed strong antagonism when combined with paclitaxel, in the human breast cancer cell line MCF7, thus having a cytoprotective effect against this anticancer agent. However, in both cases, it was observed antagonism for the corresponding nanoassemblies, due to the acid-sensitive acetonide groups in the lysosomes. The authors concluded that acid-resistant substituents should be considered in future works.

In the work of Garcia et al., the authors presented royleanone derivatives from Plectranthus spp. as a novel class of P-glycoprotein inhibitors. They reported that regarding their stability and P-gp inhibition potential, the results suggested that the formation of benzoyl esters is a more convenient approach for future derivatives with enhanced cytotoxicity. Furthermore, the authors observed that the moieties in positions six and seven of royleanones are also important for interaction with the P-pg.

Chen et al. studied the *in vivo* and *in vitro* effects of magnolol, a biphenyl extracted from *Magnolia officinali*s, on the proliferation, migration, and invasion of pancreatic cancer. They noted that magnolol, by suppressing the TGF-β signal pathway and epithelial-mesenchymal-transition (EMT), could inhibit proliferation, migration, and invasion*.* The authors concluded that magnolol might represent an effective drug for the treatment of pancreatic cancer, however its mechanism of action needs further investigation.

Overall, the works presented in this special issue provide experimental evidence and assembled scientific data, which clearly emphasize the medicinal value of natural products to fight multidrug resistant cancer cells. Nature has many bioactive molecules to offer, some of which have yet to be discovered, thus giving hope that in the future we will be able to sustainably use natural resources and find stronger and safer anti-cancer agents.

